# Carotenoid Content in Organically Produced Wheat: Relevance for Human Nutritional Health on Consumption

**DOI:** 10.3390/ijerph121114068

**Published:** 2015-11-02

**Authors:** Abrar Hussain, Hans Larsson, Ramune Kuktaite, Marie E. Olsson, Eva Johansson

**Affiliations:** 1 Department of Biosciences, COMSATS Institute of Information Technology, Sahiwal Campus, Sahiwal 57000, Pakistan; E-Mail: Abrar.Hussain@ciitsahiwal.edu.pk; 2 Department of Plant Breeding, The Swedish University of Agricultural Sciences, Box 101, SE-14 230 53 Alnarp, Sweden; E-Mails: Hans.larsson@slu.se (H.L.); Ramune.kuktaite@slu.se (R.K.); Marie.olsson@slu.se (M.O.)

**Keywords:** carotenoids, food requirements, genotypes, lutein, spring and winter wheat

## Abstract

In this study, 33 spring and winter wheat genotypes were analyzed for carotenoid content and composition. Investigated genotypes were divided into four genotype groups *i.e.*, spelt, landraces, old cultivars and primitive wheat. The results showed a high level of variation among the genotypes in amount of carotenoids in the grain with high values (around 4 mg/Kg) especially in one of the genotypes—Öland 8. Lutein was the most common carotenoid in all the investigated genotypes, contributing 70%–90% of the carotenoids in the grain. Variation in carotenoid content and composition was found not only among genotypes, but also between genotype groups and wheat type, although there is a need to analyze more genotypes to confirm the differences found between groups and types. This study showed that 40% of the daily requirements of lutein can be achieved from the genotypes with the highest lutein content (Öland 8) produced using organic farming through the average human consumption of 200 grams of wheat per day. Furthermore, this study showed, by the use of principal component analyses, an opportunity to select genotypes combining high values of certain nutritional compounds. By a further breeding and commercial production of such genotypes, the nutritional value of wheat flour for human consumption can be improved.

## 1. Introduction

Carotenoids are lipid soluble bioactive components responsible for the yellow, orange and red colors in various fruits, flowers and vegetables [1]. The carotenoids are also involved in the photosystem assembly through harvesting light and photoprotection, in helping in non-photochemical quenching and affecting the seed setting process of plants [2]. Generally, carotenoids are divided into two classes: (i) carotenes, such as α- and β*-*carotene and lycopene, which are tetraterpenoid hydrocarbons, and (ii) xanthophylls, such as lutein, β*-*cryptoxanthin and zeaxanthin, comprised of one or more oxygen-containing groups [3]. Carotenoids have been found to play an important role in the human body, e.g., α- and β-carotene have a primary function in the biosynthesis of vitamin A, an essential factor in visual functions, embryo and fetus development [4]. Lutein and zeaxanthin have been found to play an important role in promoting the health of eyes and skin and also in reducing the risk of cancer and cardiovascular diseases [5,6]. Therefore, carotenoids should be included in the human diet to promote health.

Cereals such as wheat, rice and maize are food crops consumed at high amounts, thereby being an important source of energy and proteins *per capita* around the World. Carotenoids are generally minor components of cereal grains [7], although the amount of consumption of cereals as the staple food in most human cultures might make the cereals an important carotenoid source for humans [8]. Furthermore, some cereals have even higher amounts of carotenoids in their grains than found in fruits and vegetables [9,10]. The content of carotenoids in durum wheat is also a quality character when used for pasta production where the yellow colour is seen as important [11]. Therefore, the content and composition of carotenoids in wheat grain have been evaluated [11,12,13]. Lutein and zeaxanthin have been found to be the major carotenoids in cereal grains [11,13,14]. Generally, einkorn wheat has been found to contain higher amounts of carotenoids than durum and hexaploid bread wheat [15].

The demand for organically produced food is constantly rising [16]. Consumer reasons for selecting organic food varies, although the idea that organic food contributes positively to health due to low content of chemicals and high content of nutritional compound is prevailing in some studies [17,18]. Previous studies have also indicated that organically produced foods contain higher amounts of phytochemicals, *i.e.*, carotenoids and polyphenols, than conventionally produced food [19,20]. Organically produced wheat grain has not previously been evaluated as to possible differences in carotenoid content.

The present study aimed at investigating an organically produced wheat material of diverse origin for grain carotenoid content. The same wheat material has previously been investigated for its contents of minerals, heavy metals, and tocopherols, and some organically grown wheats have been found to have high nutritious properties [21,22,23,24]. In order to better understand the options for production of nutritious wheat that can contribute to human health, the same wheat material was also investigated for carotenoid content. Contribution of the genotypes to the amounts of the various carotenoid compounds from organically produced wheat was evaluated. A second aim of the present study was thus to discuss and understand the proportion of various compounds contributing to human health in organically produced wheat grain and how nutritious wheat can be selected for further breeding and production.

## 2. Materials and Methods

### 2.1. Chemicals

Standards of *α*-carotene, β*-*carotene and lutein were obtained from Sigma-Aldrich (Schnelldorf, Germany). β*-*Cryptoxanthin and zeaxanthin were purchased from CaroteNature (Lupsingen, Switzerland. The stock solution for the standards were prepared at the concentration of 50 µg/mL of *n*-hexane and stored in the dark at −20 °C. Suitable volumes of each stock solution were used to prepare the working solutions.

### 2.2. Samples

A total of 33 wheat genotypes from diverse groups *i.e*., landraces, old cultivar, spelt and primitive wheat, were used in the present study (Table 1). The genotypes were collected from a project that aims at breeding wheat genotypes suitable for organic farming. These genotypes were grown in organic trials in Alnarp (55°39.4′ N, 13°5.2′ E, Sweden). The organic site in Alnarp has been so since 1992. The soil characteristics were: pH 7.0–7.8, organic matter 3%, clay 25%. No fertilizer or pesticides was applied. At maturity, the spikes were threshed manually and grains were kept in cool and dry room. Before analysis, the grains were lyophilized and about six grams of each grain sample was milled for 20 s to whole meal flour by a laboratory mill (Yellow line, A10, IKA-Werke, Staufen, Germany). Afterwards carotenoids were extracted from the whole meal flour.

### 2.3. Extraction by Saponification

Saponification was carried out for the extraction of carotenoids using previously described methods [25] with modifications [24]. Briefly, whole meal flour (1 g) was weighed into a screw cap Teflon tube and saponified with ethanol pyrogallol (2.5 mL, 60 g/L), sodium chloride (1 mL, 10 g/L), ethanol (1 mL, 95%) and potassium hydroxide (1 mL, 600 g/L). The tubes were placed in a 70 °C water bath for 30 min and mixed every 10 min during saponification. Afterwards, the tubes were cooled in an ice-water bath and sodium chloride and *n*-hexane/ethyl acetate (9:1) was added (7.5 mL). Then the organic layer was separated by centrifuging at 1500 rpm for 5 min. Two additional extractions were carried out by adding *n*-hexane/ethyl acetate (9:1, 5 mL) in each extraction. The organic layer was evaporated to dryness and residue was dissolved in *n*-hexane (2 mL). Each whole meal flour sample was replicated two or three times.

**Table 1:** Genotype name, type and classes of wheat used in the study, and total content of carotenoids in each sample as well as % of different carotenoid compounds found in the genotypes.

Genotype	Type	Class	Total Carotenoids (mg/kg)	% Lutein	% Zea-Xanthin	% β-Carotene	% β-Crypto-Xanthin
Aurore 2	Spring	Old cultivar	1.24	81.3	7.66	10.5	0.51
Fylgia I	Spring	Old cultivar	0.78	76.7	19.4	3.69	0.20
Lv. Dal 16 brun borst I	Spring	Landrace	1.70	89.2	9.22	1.52	0.05
Lv. Dal 16 vit	Spring	Landrace	1.24	71.5	16.9	11.1	0.46
Lv. Gotland 2	Spring	Spelt	1.76	79.1	15.3	5.32	0.23
Lv. Gotland 6	Spring	Spelt	1.61	80.9	10.8	7.87	0.39
Rival 1	Spring	Old cultivar	1.33	72.2	19.7	7.82	0.34
Öland 5	Spring	Landrace	1.02	70.2	26.4	3.24	0.13
Öland 8	Spring	Landrace	4.08	90.7	3.22	5.70	0.33
Ölands 17 borst spelt	Spring	Spelt	1.48	77.4	13.2	8.91	0.41
6356 Spelt	Winter	Spelt	1.38	76.6	11.7	11.2	0.51
Aura	Winter	Old cultivar	2.48	82.4	5.50	11.4	0.67
Brun spelt	Winter	Spelt	2.02	84.8	4.48	10.4	0.41
Hansa	Winter	Old cultivar	2.19	90.3	8.02	1.60	0.03
Holme	Winter	Old cultivar	1.86	86.5	10.4	3.02	0.14
Inntaler	Winter	Old cultivar	1.67	89.0	8.32	2.64	0.09
Jacoby 59 utan borst	Winter	Landrace	1.78	77.2	7.70	14.6	0.56
Lysh vede brun borst	Winter	Old cultivar	1.57	78.9	13.5	7.29	0.32
Mumie vete	Winter	Primitive	1.89	91.8	7.49	0.70	0.02
Oberkulmer	Winter	Spelt	2.06	90.4	4.77	4.69	0.18
Odin	Winter	Old cultivar	1.77	84.1	9.93	5.64	0.30
Olympia	Winter	Landrace	2.14	92.1	6.69	1.15	0.03
Oster burgsdorfer	Winter	Spelt	1.62	83.6	9.65	6.33	0.39
Rauweizen	Winter	Primitive	2.03	86.9	12.1	0.98	0.00
Robur	Winter	Old cultivar	1.55	80.8	10.2	8.61	0.33
Röd Emmer	Winter	Primitive	1.32	80.6	18.4	0.99	0.03
Schwaben korn	Winter	Spelt	2.23	88.4	4.22	7.17	0.24
Schweiz	Winter	Spelt	2.50	88.6	3.12	8.01	0.31
Spelt Ustakket	Winter	Spelt	2.16	87.9	6.24	5.73	0.18
Spelt vete gotland	Winter	Spelt	1.95	82.8	7.45	9.38	0.41
Svale	Winter	Old cultivar	1.96	89.8	9.05	1.16	0.01
Svart emmer	Winter	Primitive	1.80	89.6	8.42	1.89	0.08
T.polonicum	Winter	Primitive	0.94	80.9	15.1	3.88	0.13

### 2.4. HPLC Analysis

Carotenoid compounds were separated by a normal phase HPLC method [14] with some modifications. Separation was achieved by a 250 × 4.6 mm i.d., 5 µm particle size LUNA Silica column Phenomenex (Phenomenex, Torrance, CA, USA). The mobile phase was *n*-hexane/isopropyl alcohol (5%) and flow rate was 1.5 mL/min. Detection was achieved by PDA detector and peaks were detected at 450 nm. The volume of each injection was 100 µL. Carotenoid compounds were identified by their particular spectra and their retention time was compared with respective standards. Also, the column was cleaned after every 12 injections with a solution of 10% (v/v) isopropyl alcohol and *n*-hexane.

### 2.5. Data Analysis

Principal component analysis (PCA), Analysis of Variance (ANOVA), Spearman rank correlation and variances were calculated by using the Statistical Analysis System [26]. Mean comparisons after ANOVA were carried out using the Duncan multiple range test. Genotypes were also ranked for content of various compounds and thereafter regression analysis was performed using SAS in order to explain contribution of genotype to the variation of the respective compound.

## 3. Results and Discussion

### 3.1. Genetic Variation in Content of Carotenoids

**Figure 1:**
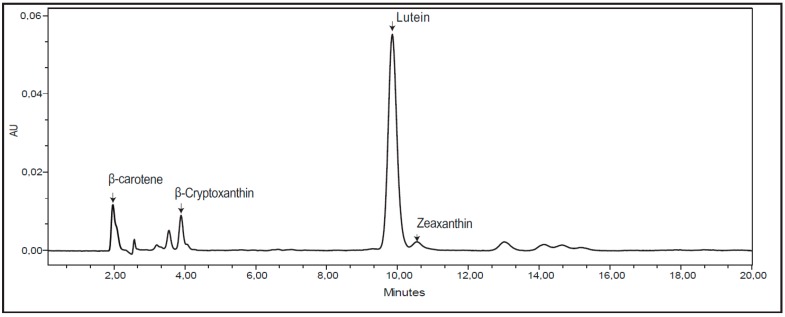
Representative chromatogram of the genotype Öland 8.

The total content of carotenoids in the investigated wheat material ranged from 0.94 to 4.08 mg/kg (Table 1; separate values for all analyzed samples and compounds are given in Supplemental Table S1). These values correspond well to what has been measured in wheat materials in previous investigations (1.27–13.6 mg/kg) [11,15,27,28,29]. Generally, much higher contents of carotenoids have been reported in einkorn wheat (4–14 mg/Kg) than in durum (3–6 mg/kg) and bread wheat (1–3 mg/kg) [11,15,27,29]. The genotype with the highest total amount of carotenoids in the present investigation is a hexaploid landrace of spring wheat originating from the Swedish island of Öland, named Öland 8 (Table 1, Figure 1). No einkorn or durum wheats were investigated in the present study. In principle, four different types of carotenoids, β-carotene, β-cryptoxanthin, lutein and zeaxantin, were found in all the investigated genotypes, except for one genotype, Rauweizen, a primitive winter wheat (*T. turgidum*), lacking content of β-cryptoxanthin (results not shown). As compared to other studies, similarities but also differences as related to the carotenoid composition in the present study have been reported. Previous studies have reported similar carotenoid compounds as found in the present study [27], the presence of the same compounds in einkorn wheat but only presence of lutein and β-carotene in common wheat [11], lutein, α-carotene and β-carotene in bread wheat [15], lutein, β-carotene and zeaxantin in soft wheat [28] and the same compound plus α-carotene in spring wheat [30], while the winter wheat lacked α-carotene [30], lutein and zeaxanthin in bread, durum and einkorn wheat [29]. The found similarities and differences reported among the different studies, might be related to extraction methods and separation methods used, but also to variation in different wheat materials, as these variables have been found important also in previous investigations on bioactive compounds [31]. However, all studies, including ours, have reported lutein as being the predominant carotenoid present in wheat. In our material, content of lutein was 70.2%–92.1% of the total carotenoids (Table 1). Previous studies have reported lutein to correspond to from around 70% of the total carotenoids up to levels of above 97% [11,15,27,28,29,30]. In our study, the second most common carotenoid was for most genotypes zeaxanthin, 3.1%–26.4% of the total amount of carotenoids (Table 1). Previous studies report from no zeaxanthin at all in wheat to up to around 25% [11,15,27,28,29,30]. Thus, the present study showed a large variation in content of zeaxanthin in the investigated genotypes and in several genotypes relatively high values of zeaxanthin, compared to previous studies. For most genotypes in the present study β-carotene was the third most prevalent carotenoid and for some genotypes even the one with the second highest value, 0.70%–14.6% of the carotenoids (Table 1). The presence of β-carotene was also reported in most other studies on wheat, however in the lower range compared to what was found in the present study [11,15,27,28,29,30]. The range of β-cryptoxanthin in the wheat in the present study was 0%–0.67%, thereby being the carotenoid showing lowest amounts of those found in the present material (Table 1). Only a few other studies have reported the presence of β-cryptoxanthin in wheat, besides for einkorn [11,27]. Thus for the wheat in the present study, a rather large variation was seen in the content of found carotenoids in the different genotypes compared to previous investigations and also genotypes with rather high values of the different found compounds were present.

### 3.2. Variation among Genotype Groups

Significant differences were found among the genotype groups for the concentration of carotenoids in organically produced whole meal flour (Table 2). Landraces showed significantly higher amounts of total carotenoids. The reason for the high amount of total carotenoids was the high amount of lutein in the landrace group (Table 2). The amount of zeaxanthin was the highest in the whole meal flour of the primitive group as compared to other investigated genotype groups (Table 2). The spelt group showed the highest amount of β-carotene and β-cryptoxanthin as compared to all other investigated genotype groups in the present study (Table 2). Although significant differences were found among the different genotype groups, large variations were also found within the genotype groups indicating that the genotype in itself can be as important as the genotype group for content of different carotenoids in the wheat grain. Furthermore, the number of genotypes in each of the different genotype groups was rather small in the present investigation, suggesting a larger number of genotypes to be analyzed before conclusions on variations of specific carotenoids can be drawn. Previous studies have shown variation in total amounts of carotenoids between einkorn, durum and common wheat [29] and presence and absence of certain types of carotenoids in the same type of wheat [11].

**Table 2:** Variation among genotype groups for different carotenoids content (mg/kg) in organically produced wheat grain.

Genotype Group	Lutein	Zeaxantin	β-Carotene	β-Cryptoxantin	Total
Spelt	1.60 ^b^	0.14 ^c^	0.14 ^a^	0.006 ^a^	1.88 ^b^
Landrace	1.70 ^a^	0.17 ^b^	0.12 ^b^	0.005 ^b^	1.99 ^a^
Old cultivar	1.42 ^c^	0.17 ^b^	0.09 ^c^	0.004 ^c^	1.69 ^c^
Primitive	1.54 ^b^	0.19 ^a^	0.02 ^d^	0.001 ^d^	1.75 ^c^

Note: Mean values followed by the same superscript letter do not differ significantly from each other at *p* < 0.05 by using Duncan’s multiple range test.

### 3.3. Variation Between Spring and Winter Wheat

In the present study, winter wheat showed a higher total carotenoid content (1.88 mg/kg) than spring wheat (1.61 mg/kg), mainly as a result of its higher lutein content (Table 3). Spring wheat showed a significantly higher amount of zeaxanthin and β-cryptoxanthin than winter wheat (Table 3). No significant difference was found between spring and winter wheat for the concentration of β-carotene in whole meal flour (Table 3). These results differ from those in a previous study showing a higher total carotenoid content in spring wheat (3.52 mg/kg) than in winter wheat (2.42 mg/kg) [30]. In the mentioned previous study, the difference between spring and winter wheat was fully explained by differences in content of lutein between the spring and winter wheat [30], and by the fact that α-carotene was found in spring wheat but not in winter wheat. In both the present study and the previous study [30], the number of investigated samples was rather small (10 + 23 and 5 + 6, respectively) and the variation within the various groups rather high, which might indicate that the found differences between spring and winter type is more related to the selection of genotypes than to the type of wheat.

**Table 3:** Difference between spring and winter for different carotenoids content (mg/kg) in organically produced wheat grain.

Wheat Type	Lutein	Zeaxantin	β-Carotene	β-Cryptoxantin	Total
Spring	1.31 ^b^	0.19 ^a^	0.10 ^a^	0.005 ^a^	1.61 ^b^
Winter	1.62 ^a^	0.16 ^b^	0.10 ^a^	0.004 ^b^	1.88 ^a^

Note: Mean values followed by the same superscript letter do not differ significantly from each other at *p* < 0.05 by using Duncans multiple range test.

### 3.4. Human Requirement of Various Carotenoid Compounds

Carotenoids are known as being important in the human diet, mostly as vitamin A, where a deficit may contribute to an increase in childhood death [32]. Different recommendations of daily intake of vitamin A are prevailing, *i.e*., 0.6 mg/day [32] or a plasma retinol concentration of 0.70 µmol/L [33]. Only lipophilic carotenoids, such as the α-carotene and β-carotenes and β-cryptoxanthin have been shown to have pro-vitamin A activity, thereby contributing to vitamin A formation [33]. Various conversion factors have been used for calculations of needs of pro-vitamin A carotenoids; from 1:6 for vitamin A:β-carotene and 1:12 for vitamin A:all other provitamin A carotenoids, to 1:14 and 1:28 for the same carotenoids [33]. Besides carotenoid type and conversion factors, fat in the diet plays an essential role for the uptake and bioavailability of carotenoids as precursors for vitamin A. As wheat in general, including the diverse material in the present study, is low in carotenoids, especially compared to animal products [33], but also to certain fruits and berries [32], such as e.g., sea buckthorn berries [34] and rose hips [35], and also have a low level of carotenoids with provitamin A activity, wheat cannot be considered an interesting source of vitamin A.

The carotenoid types with the highest amount in the wheat in the present study as well as in previous ones [11,15,27,28,29,30] were lutein and zeaxanthin. These carotenoids, and particularly lutein, have been associated with macular pigment concentrations in the eye retina and some correlations have also been described with prevention of cardiovascular diseases and cancer [36,37]. The major contributors to dietary lutein have been reported to be green leafy vegetables, e.g., spinach, while corn products have been reported the major contributors of dietary zeaxanthin [38]. Recommendations are available of serum lutein concentrations of 0.6 to 1.05 µmol/L (350–600 µg/L) to secure good visual function and eventually protection against other chronic diseases [36]. A significant relationship between dietary intake of lutein + zeaxanthin and serum content of lutein + zeaxanthin have been reported (r = 0.21) [39]. An average intake of lutein + zeaxanthin of 1101 ± 838 µg/day has been reported and of serum concentrations of 0.36 µmol/L have been found in a population from Indianapolis (IN, USA) [39]. Lutein is available as supplements for human consumption and the consumption of lutein-containing supplements is increasing, although no clear positive effects of intake of lutein supplemented food have been demonstrated [38].

### 3.5. Importance of Organically Produced Wheat as a Source of Lutein

Mean consumption of wheat flour is known to be 200 g per person per day and has been used in previous studies to calculate if wheat is an important source for the intake of various compounds [21]. As to the above discussion related to intake of lutein + zeaxanthin, the required average intake is 1.83 mg/day in order to reach an average serum concentration of 0.6 µmol/L. Based on the assumption that the average intake of wheat is 200 g per person and day, the average intake of lutein + zeaxanthin from the studied wheat material would be 0.15–0.77 mg/day (Table 4). Thus, from consumption of 200 g of Öland 8 (the genotype with the highest lutein content) per day, the average person will receive 40% lutein + zeaxanthin as related to average daily requirements.

**Table 4:** Intake per day of lutein+zeaxanthin from an average consumption of 200 g wheat per day.

Genotype	Type	Class	Lutein + Zeaxanthin Intake from 200 g Wheat per Day (mg/day)
Aurore 2	Spring	Old cultivar	0.22
Fylgia I	Spring	Old cultivar	0.15
Lv. Dal 16 brun borst I	Spring	Landrace	0.34
Lv. Dal 16 vit	Spring	Landrace	0.22
Lv. Gotland 2	Spring	Spelt	0.33
Lv. Gotland 6	Spring	Spelt	0.30
Rival 1	Spring	Old cultivar	0.24
Öland 5	Spring	Landrace	0.20
Öland 8	Spring	Landrace	0.77
Ölands 17 borst spelt	Spring	Spelt	0.27
6356 Spelt	Winter	Spelt	0.24
Aura	Winter	Old cultivar	0.44
Brun spelt	Winter	Spelt	0.36
Hansa	Winter	Old cultivar	0.43
Holme	Winter	Old cultivar	0.36
Inntaler	Winter	Old cultivar	0.32
Jacoby 59 utan borst	Winter	Landrace	0.30
Lysh vede brun borst	Winter	Old cultivar	0.29
Mumie vete	Winter	Primitive	0.37
Oberkulmer	Winter	Spelt	0.39
Odin	Winter	Old cultivar	0.33
Olympia	Winter	Landrace	0.42
Oster burgsdorfer	Winter	Spelt	0.30
Rauweizen	Winter	Primitive	0.40
Robur	Winter	Old cultivar	0.28
Röd Emmer	Winter	Primitive	0.26
Schwaben korn	Winter	Spelt	0.41
Schweiz	Winter	Spelt	0.46
Spelt Ustakket	Winter	Spelt	0.41
Spelt vete gotland	Winter	Spelt	0.35
Svale	Winter	Old cultivar	0.39
Svart emmer	Winter	Primitive	0.35
T.polonicum	Winter	Primitive	0.18

### 3.6. Proportion of Importance of Different Compounds from Organically Produced Wheat for Human Health

The wheat material in the present study have in this and previous studies [21,22,23,24] been investigated for a number of compounds (carotenoids, tocopherols, minerals and heavy metals) of which the intake is related to human health. For these studies we have selected a wheat material of broad genetic background although we did not include either einkorn nor durum wheat. In general we obtained a large variation in the compounds studied from the present material. While ranking the wheat genotypes based on their content of various compounds and thereafter calculating the proportion of the contribution of variation by the genotypes [40], the influence of the genotypes on the variation of the respective compounds was in general found to be above 90%, although lower values of 40%–50% were found for some of the heavy metals (Table 5).

**Table 5:** Percentage of explanation (obtained through R-square from simple linear regression) of the evaluated compounds on ranked genotypes.

Compound	Genotype
Lutein	79.9
Zeaxanthin	92.0
β-Carotene	93.1
β-Cryptoxanthin	86.1
α-Tocopherol	96.3
β-Tocopherol	92.8
α -Tocotrienol	61.9
β-Tocotrienol	97.5
Iron	93.6
Potassium	89.8
Magnesium	95.9
Sodium	88.1
Phosphorus	89.8
Zinc	97.4
Copper	98.6
Cadmium	96.7
Cobalt	76.5
Crom	46.1
Nickel	45.8
Pb	62.9

**Figure 2:**
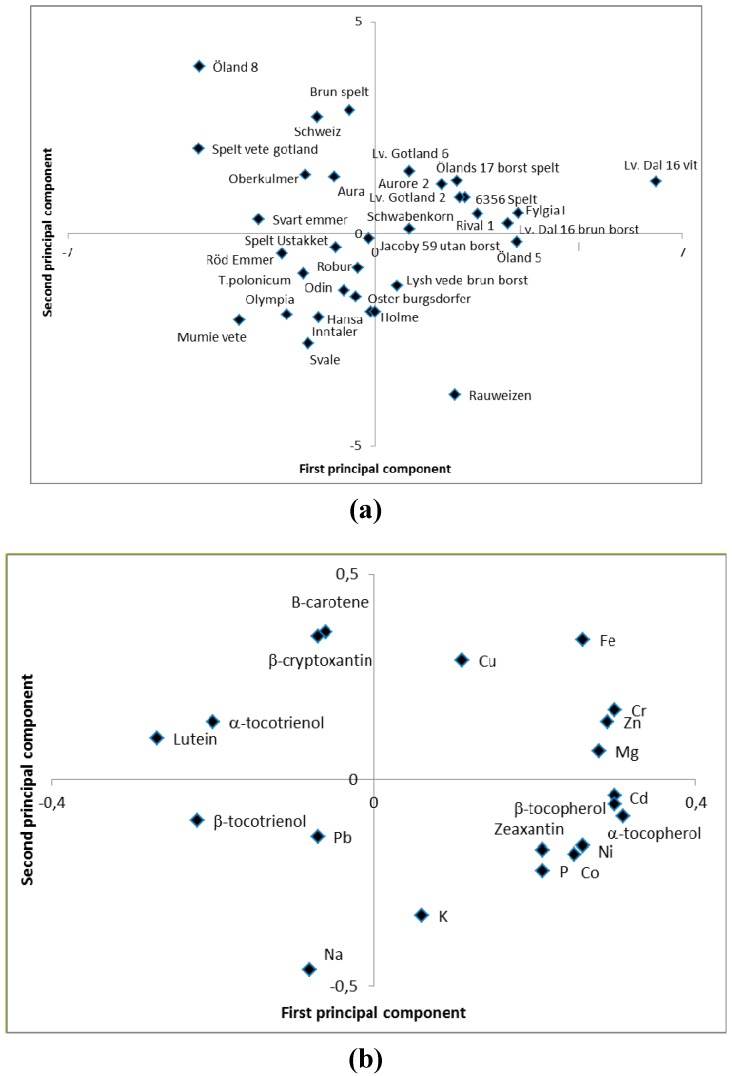
Loading (**a**) and score (**b**) plot from principal component analysis of compounds (carotenoids, tocopherols, minerals and heavy metals) in wheat of a diverse background. First principal component explained 24.8% of the variation while the second principal component explained 14.1% of the variation.

Principal component analyses of the data showed that most of the carotenoids and the tocotrienols co-varied in the material with negative values on the first principal component and positive values on the second principal component (Figure 2a). A negative association was found between the tocotrienols and the tocopherols (Figure 2a). Positive values on both the principal component analyses were found for several of the minerals, and among those for iron and zinc (Figure 2a). Most of the heavy metals showed positive values on the first principal component and negative values on the second principal component (Figure 2a). Thus, selections of genotypes in relation to breeding and/or production of material with high nutritional value can be carried out based on the principal component analyses. In principal, for such a selection, genotypes with a positive value on the second principal component should be selected, those with a negative value on the first principal component for high levels of lutein and tocotrienols and those with a positive value on the first principal component for high levels of iron and zinc (Figure 2b).

## 4. Conclusions

The wheat materials in the present study showed a large variation in amount of carotenoids in their grain, with some genotypes, e.g., Öland 8, showing high values of around 4 mg/kg. The most common carotenoid in all investigated genotypes was lutein, contributing with 70%–90% of the amount of carotenoids in the grains. The investigated content of various carotenoids varied related both genotype group and wheat type although due to the limited amount of genotypes investigated, such relationships need further confirmation before clearcut conclusions can be drawn. The major carotenoid compound in wheat, lutein, is important in human consumption due to its contribution to health of eyes but it also in prevention of cancer and cardiovascular diseases. The wheat with the highest amount of lutein in the grain has the potential to contribute with 40% of the daily requirement of lutein for the average human if 200 g of wheat is consumed daily, an amount of wheat consumption known to be the average human consumption. Einkorn wheat genotypes with high lutein content can contribute to more than full daily requirement of lutein. Principal component analyses can be applied to select genotypes for further breeding and production of wheat with potentially high nutritional value, although high values of all investigated nutritional compounds were not found in any of the genotypes in the present study.

## Supplementary Files

Supplementary File 1
