# Supplementary material for: Carotenoid Content in Organically Produced Wheat: Relevance for Human Nutritional Health on Consumption
Source: Int J Environ Res Public Health. 2015 Nov 2;12(11):14068–83. doi: 10.3390/ijerph121114068 (PMC4661633; doi:10.3390/ijerph121114068)
Supplement: Supplementary File 1 [file ijerph-12-14068-s001.pdf]

Supplementary Information

Carotenoid Content in Organically Produced Wheat: Relevance for Human Nutritional Health on Consumption

Table S1. Content of different carotenoids and total carotenoid content in analyzed samples of wheat.

Genotype	Type	Class	β-Caroten	β-Cryptoxantin	Lutein	Zeaxantin	Total mg/kg
Aurore 2	s	o.c.	0.127	0.0061	1.001	0.100	1.234
	s	o.c.	0.135	0.0065	1.021	0.090	1.252
Fylgia I	s	o.c	0.035	0.0019	0.591	0.140	0.769
	s	o.c	0.023	0.0012	0.610	0.164	0.7984
Lv. Dal 16 brun borst I	s	l.	0.025	0.0009	1.530	0.162	1.718
	s	l.	0.027	0.0008	1.509	0.152	1.689
Lv. Dal 16 vit	s	l.	0.144	0.0060	0.883	0.208	1.241
	s	l.	0.131	0.0054	0.890	0.211	1.238
Lv. Gotland 2	s	s.p.	0.104	0.0049	1.443	0.275	1.827
	s	s.p.	0.083	0.0033	1.344	0.264	1.694
Lv. Gotland 6	s	s.p.	0.123	0.0061	1.405	0.197	1.731
	s	s.p.	0.132	0.0067	1.428	0.198	1.764
	s	s.p.	0.126	0.0062	1.074	0.129	1.336
Rival 1	s	o.c.	0.111	0.0050	0.960	0.266	1.341
	s	o.c.	0.099	0.0040	0.910	0.242	1.255
	s	o.c.	0.101	0.0044	1.004	0.275	1.384
Öland 5	s	l.	0.035	0.0015	0.717	0.271	1.025
	s	l.	0.031	0.0012	0.713	0.267	1.012
Öland 8	s	l.	0.228	0.0134	3.705	0.140	4.086
	s	l.	0.237	0.0139	3.700	0.123	4.074
Ölands 17 borst spelt	s	s.p.	0.138	0.0060	1.160	0.197	1.501
	s	s.p.	0.126	0.0061	1.131	0.195	1.457

Table S1. *Cont.*

Genotype	Type	Class	β-Caroten	β-Cryptoxantin	Lutein	Zeaxantin	Total mg/kg
6356 Spelt	w	s.p.	0.160	0.0070	1.034	0.147	1.348
	w	s.p.	0.138	0.0069	1.106	0.169	1.419
	w	s.p.	0.165	0.0074	1.026	0.166	1.364
Aura	w	o.c.	0.268	0.0163	2.098	0.139	2.521
	w	o.c.	0.299	0.0168	1.986	0.134	2.436
Brun spelt	w	s.p.	0.209	0.0080	1.680	0.070	1.967
	w	s.p.	0.208	0.0086	1.740	0.111	2.067
Hansa	w	o.c.	0.037	0.0008	2.198	0.189	2.426
	w	o.c.	0.034	0.0007	1.964	0.186	2.185
	w	o.c.	0.035	0.0007	1.783	0.153	1.970
Holme	w	o.c.	0.057	0.0029	1.717	0.207	1.986
	w	o.c.	0.054	0.0021	1.504	0.164	1.724
	w	o.c.	0.057	0.0027	1.594	0.204	1.859
Inntaler	w	o.c.	0.043	0.0017	1.455	0.138	1.638
	w	o.c.	0.042	0.0013	1.466	0.138	1.647
	w	o.c.	0.047	0.0016	1.528	0.141	1.717
Jacoby 59 utan borst	w	l.	0.223	0.0092	1.484	0.146	1.862
	w	l.	0.264	0.0102	1.304	0.125	1.703
	w	l.	0.294	0.0103	1.340	0.141	1.785
Lysh vede brun borst	w	o.c.	0.108	0.0052	1.229	0.216	1.558
	w	o.c.	0.105	0.0048	1.222	0.216	1.548
	w	o.c.	0.129	0.0051	1.264	0.206	1.604
Mumie vete	w	p.	0.013	0.0003	1.591	0.129	1.734
	w	p.	0.015	0.0004	1.797	0.149	1.962
	w	p.	0.012	0.0004	1.802	0.144	1.959
Oberkulmer	w	s.p.	0.103	0.0040	1.983	0.099	2.188
	w	s.p.	0.086	0.0033	1.853	0.102	2.044
	w	s.p.	0.102	0.0039	1.754	0.094	1.954

Table S1. *Cont.*

Genotype	Type	Class	β-Caroten	β-Cryptoxantin	Lutein	Zeaxantin	Total mg/kg
Odin	w	o.c.	0.099	0.0054	1.671	0.214	1.990
	w	o.c.	0.096	0.0053	1.385	0.148	1.634
	w	o.c.	0.105	0.0054	1.418	0.166	1.695
Olympia	w	l.	0.026	0.0008	1.939	0.142	2.109
	w	l.	0.025	0.0005	1.918	0.143	2.086
	w	l.	0.023	0.0004	2.058	0.144	2.225
Oster burgsdorfe	w	s.p.	0.090	0.0058	1.399	0.158	1.653
	w	s.p.	0.087	0.0057	1.335	0.151	1.579
	w	s.p.	0.130	0.0074	1.325	0.159	1.621
Rauweizen	w	p.	0.019	0.0000	1.782	0.260	2.061
	w	p.	0.024	0.0000	1.699	0.225	1.948
	w	p.	0.018	0.0000	1.818	0.252	2.088
Robur	w	o.c.	0.114	0.0043	1.217	0.155	1.491
	w	o.c.	0.137	0.0051	1.231	0.165	1.538
	w	o.c.	0.149	0.0060	1.314	0.158	1.627
Röd Emmer	w	p.	0.012	0.0000	1.158	0.278	1.449
	w	p.	0.014	0.0006	1.060	0.232	1.307
	w	p.	0.013	0.0006	0.973	0.217	1.203
Schwaben korn	w	s.p.	0.171	0.0056	2.041	0.096	2.314
	w	s.p.	0.154	0.0050	1.888	0.089	2.136
	w	s.p.	0.155	0.0051	1.993	0.098	2.252
Schweiz	w	s.p.	0.182	0.0068	2.408	0.083	2.680
	w	s.p.	0.218	0.0090	2.019	0.073	2.319
Spelt Ustakket	w	s.p.	0.107	0.0031	1.773	0.117	2.000
	w	s.p.	0.129	0.0043	1.954	0.148	2.236
	w	s.p.	0.137	0.0043	1.977	0.140	2.258

Table S1. *Cont.*

Genotype	Type	Class	β-Caroten	β-Cryptoxantin	Lutein	Zeaxantin	Total mg/kg
Spelt vete gotland	w	s.p.	0.175	0.0078	1.499	0.135	1.817
	w	s.p.	0.172	0.0080	1.683	0.143	2.006
	w	s.p.	0.203	0.0082	1.667	0.159	2.036
Svale	w	o.c.	0.027	0.0000	1.806	0.184	2.016
	w	o.c.	0.022	0.0000	1.778	0.189	1.989
	w	o.c.	0.019	0.0006	1.698	0.161	1.879
Svart emmer	w	p.	0.035	0.0015	1.550	0.146	1.732
	w	p.	0.034	0.0015	1.550	0.147	1.732
	w	p.	0.034	0.0011	1.745	0.163	1.942
T.polonicum	w	p.	0.048	0.0016	0.886	0.172	1.107
	w	p.	0.024	0.0008	0.629	0.112	0.766
Aurore 2	s	o.c.	0.127	0.0061	1.001	0.100	1.234
	s	o.c.	0.135	0.0065	1.021	0.090	1.252
Fylgia I	s	o.c.	0.035	0.0019	0.591	0.140	0.769
	s	o.c.	0.023	0.0012	0.610	0.164	0.7984
Lv. Dal 16 brun borst I	s	l.	0.025	0.0009	1.530	0.162	1.718
	s	l.	0.027	0.0008	1.509	0.152	1.689
Lv. Dal 16 vit	s	l.	0.144	0.0060	0.883	0.208	1.241
	s	l.	0.131	0.0054	0.890	0.211	1.238
Lv. Gotland 2	s	s.p.	0.104	0.0049	1.443	0.275	1.827
	s	s.p.	0.083	0.0033	1.344	0.264	1.694
Lv. Gotland 6	s	s.p.	0.123	0.0061	1.405	0.197	1.731
	s	s.p.	0.132	0.0067	1.428	0.198	1.764
	s	s.p.	0.126	0.0062	1.074	0.129	1.336
Rival 1	s	o.c.	0.111	0.0050	0.960	0.266	1.341
	s	o.c.	0.099	0.0040	0.910	0.242	1.255
	s	o.c.	0.101	0.0044	1.004	0.275	1.384

Table S1. *Cont.*

Genotype	Type	Class	β-Caroten	β-Cryptoxantin	Lutein	Zeaxantin	Total mg/kg
Öland 5	s	l.	0.035	0.0015	0.717	0.271	1.025
	s	l.	0.031	0.0012	0.713	0.267	1.012
Öland 8	s	l.	0.228	0.0134	3.705	0.140	4.086
	s	l.	0.237	0.0139	3.700	0.123	4.074
Ölands 17 borst spelt	s	s.p.	0.138	0.0060	1.160	0.197	1.501
	s	s.p.	0.126	0.0061	1.131	0.195	1.457
6356 Spel	w	s.p.	0.160	0.0070	1.034	0.147	1.348
	w	s.p.	0.138	0.0069	1.106	0.169	1.419
	w	s.p.	0.165	0.0074	1.026	0.166	1.364
Aura	w	o.c.	0.268	0.0163	2.098	0.139	2.521
	w	o.c.	0.299	0.0168	1.986	0.134	2.436
Brun spelt	w	s.p.	0.209	0.0080	1.680	0.070	1.967
	w	s.p.	0.208	0.0086	1.740	0.111	2.067
Hansa	w	o.c.	0.037	0.0008	2.198	0.189	2.426
	w	o.c.	0.034	0.0007	1.964	0.186	2.185
	w	o.c.	0.035	0.0007	1.783	0.153	1.970
Holme	w	o.c.	0.057	0.0029	1.717	0.207	1.986
	w	o.c.	0.054	0.0021	1.504	0.164	1.724
	w	o.c.	0.057	0.0027	1.594	0.204	1.859
Inntaler	w	o.c.	0.043	0.0017	1.455	0.138	1.638
	w	o.c.	0.042	0.0013	1.466	0.138	1.647
	w	o.c.	0.047	0.0016	1.528	0.141	1.717
Jacoby 59 utan borst	w	l.	0.223	0.0092	1.484	0.146	1.862
	w	l.	0.264	0.0102	1.304	0.125	1.703
	w	l.	0.294	0.0103	1.340	0.141	1.785
Lysh vede brun borst	w	o.c.	0.108	0.0052	1.229	0.216	1.558
	w	o.c.	0.105	0.0048	1.222	0.216	1.548
	w	o.c.	0.129	0.0051	1.264	0.206	1.604

Table S1. *Cont.*

Genotype	Type	Class	β-Caroten	β-Cryptoxantin	Lutein	Zeaxantin	Total mg/kg
Mumie vete	w	p.	0.013	0.0003	1.591	0.129	1.734
	w	p.	0.015	0.0004	1.797	0.149	1.962
	w	p.	0.012	0.0004	1.802	0.144	1.959
Oberkulmer	w	s.p.	0.103	0.0040	1.983	0.099	2.188
	w	s.p.	0.086	0.0033	1.853	0.102	2.044
	w	s.p.	0.102	0.0039	1.754	0.094	1.954
Odin	w	o.c.	0.099	0.0054	1.671	0.214	1.990
	w	o.c.	0.096	0.0053	1.385	0.148	1.634
	w	o.c.	0.105	0.0054	1.418	0.166	1.695
Olympia	w	l.	0.026	0.0008	1.939	0.142	2.109
	w	l.	0.025	0.0005	1.918	0.143	2.086
	w	l.	0.023	0.0004	2.058	0.144	2.225
Oster burgsdorfer	w	s.p.	0.090	0.0058	1.399	0.158	1.653
	w	s.p.	0.087	0.0057	1.335	0.151	1.579
	w	s.p.	0.130	0.0074	1.325	0.159	1.621
Rauweizen	w	p.	0.019	0.0000	1.782	0.260	2.061
	w	p.	0.024	0.0000	1.699	0.225	1.948
	w	p.	0.018	0.0000	1.818	0.252	2.088
Robur	w	o.c.	0.114	0.0043	1.217	0.155	1.491
	w	o.c.	0.137	0.0051	1.231	0.165	1.538
	w	o.c.	0.149	0.0060	1.314	0.158	1.627
Röd Emmer	w	p.	0.012	0.0000	1.158	0.278	1.449
	w	p.	0.014	0.0006	1.060	0.232	1.307
	w	p.	0.013	0.0006	0.973	0.217	1.203
Schwaben korn	w	s.p.	0.171	0.0056	2.041	0.096	2.314
	w	s.p.	0.154	0.0050	1.888	0.089	2.136
	w	s.p.	0.155	0.0051	1.993	0.098	2.252

Table S1. *Cont.*

Genotype	Type	Class	β-Caroten	β-Cryptoxantin	Lutein	Zeaxantin	Total mg/kg
Schweiz	w	s.p.	0.182	0.0068	2.408	0.083	2.680
	w	s.p.	0.218	0.0090	2.019	0.073	2.319
Spelt Ustakket	w	s.p.	0.107	0.0031	1.773	0.117	2.000
	w	s.p.	0.129	0.0043	1.954	0.148	2.236
	w	s.p.	0.137	0.0043	1.977	0.140	2.258
Spelt vete gotland	w	s.p.	0.175	0.0078	1.499	0.135	1.817
	w	s.p.	0.172	0.0080	1.683	0.143	2.006
	w	s.p.	0.203	0.0082	1.667	0.159	2.036
Svale	w	o.c.	0.027	0.0000	1.806	0.184	2.016
	w	o.c.	0.022	0.0000	1.778	0.189	1.989
	w	o.c.	0.019	0.0006	1.698	0.161	1.879
Svart emmer	w	p.	0.035	0.0015	1.550	0.146	1.732
	w	p.	0.034	0.0015	1.550	0.147	1.732
	w	p.	0.034	0.0011	1.745	0.163	1.942
T.polonicum	w	p.	0.048	0.0016	0.886	0.172	1.107
	w	p.	0.024	0.0008	0.629	0.112	0.766

© 2015 by the authors; licensee MDPI, Basel, Switzerland. This article is an open access article distributed under the terms and conditions of the Creative Commons Attribution license (http://creativecommons.org/licenses/by/4.0/).
